# Disruptions in RNA Splicing: A Key Regulator of Cognitive Impairment in Perioperative Neurocognitive Disorders

**DOI:** 10.1007/s12264-026-01630-5

**Published:** 2026-05-11

**Authors:** Xu-An Wang, Qing-Ya Wu, Xiao-Yu Shi, Xiao-Peng Wang, Yan-Yan Gao, Wei-Wei Zhang, Yi-Sheng Lu, Dan Liu, Ling-Qiang Zhu, Hua Zheng

**Affiliations:** 1 Department of Anesthesiology, Shanxi Bethune Hospital, Shanxi Academy of Medical Sciences, Third Hospital of Shanxi Medical University, Tongji Shanxi Hospital, Taiyuan, 030032 China; 2 Department of Anesthesiology, Hubei Key Laboratory of Geriatric Anesthesia and Perioperative Brain Health, and Wuhan Clinical Research Center for Geriatric Anesthesia, Tongji Hospital, Tongji Medical College, Huazhong University of Science and Technology, Wuhan, 430030 China; 3 Department of Conservative Dentistry and Endodontics, Shanxi Province Key Laboratory of Oral Diseases Prevention and New Materials, Shanxi Medical University School and Hospital of Stomatology, Taiyuan, 030001 China; 4 Department of Physiology, School of Basic Medicine, Tongji Medical College, Huazhong University of Science and Technology, Wuhan, 430030 China; 5 Department of Medical Genetics, School of Basic Medicine, Tongji Medical College, Huazhong University of Science and Technology, Wuhan, 430030 China; 6 Department of Pathophysiology, School of Basic Medicine, Tongji Medical College, Huazhong University of Science and Technology, Wuhan, 430030 China

**Keywords:** Perioperative neurocognitive disorders, RNA splicing, Neuroinflammation, Synaptic plasticity, BDNF, Oxidative stress, Mitochondrial dysfunction

## Abstract

Perioperative neurocognitive disorders (PND) encompass a spectrum of cognitive impairments that emerge during the perioperative period. The pathogenesis of PND is multifactorial, involving complex interactions between neuroinflammation, impairment of synaptic plasticity, dysregulation of brain-derived neurotrophic factor (BDNF) signaling, oxidative stress, and mitochondrial dysfunction. RNA splicing is increasingly recognized as a critical player in nervous system function and dysfunction. In this review, we explore the role of RNA splicing in modulating the major pathogenic mechanisms of PND. We highlight how aberrant splicing events promote neuroinflammation by altering the expression of pro-inflammatory genes, disrupt synaptic plasticity by modifying the profiles of synaptic proteins and neurotransmitter receptors, and impair the expression and function of BDNF. Additionally, RNA splicing abnormalities exacerbate oxidative stress and mitochondrial dysfunction, further amplifying neuronal damage. By elucidating the intricate interactions between RNA splicing and these core pathological processes, this review reveals the close association between RNA splicing dysregulation and the molecular underpinnings of PND, and highlights its potential regulatory role in PND pathogenesis.

## Introduction

Perioperative neurocognitive disorders (PND) represent a spectrum of cognitive impairments that emerge in the context of anesthesia and surgery, encompassing conditions such as postoperative delirium (POD) and postoperative neurocognitive disorder [1]. The incidence of PND is substantially higher in elderly patients, with reported rates varying from 10% to over 65%, making advanced age a primary risk factor. PND is clinically significant due to the strong association with adverse outcomes, including increased postoperative mortality, prolonged hospital stays, diminished long-term quality of life, and a greater need for custodial care, thereby imposing substantial socioeconomic burdens [2]. Beyond age, the pathogenesis of PND is multifactorial, involving elements such as surgical trauma, anesthetic agent exposure, pre-existing cognitive vulnerability, lower educational attainment, perioperative pain, anticholinergic medications, and comorbidities including mental health disorders and sleep disturbances [3, 4]. Although PND most commonly manifests acutely within the first postoperative week, its sequelae can persist for months or even years, and emerging evidence suggests it may accelerate the progression to enduring cognitive decline and neurodegenerative diseases such as Alzheimer's disease [5]. The precise pathophysiological mechanisms of PND remain incompletely elucidated, underscoring the critical need for continued investigation into its molecular foundations.

RNA splicing is an indispensable process in eukaryotic gene expression, responsible for the precise removal of introns from pre-messenger RNA (pre-mRNA) and the ligation of exons to produce mature mRNA transcripts. This process occurs through two primary mechanisms: constitutive splicing, which yields a single mRNA variant, and alternative splicing, which allows for the selective inclusion or exclusion of exons to generate multiple distinct transcripts from a single gene, thereby vastly expanding proteomic diversity and functional specificity [6]. The nervous system is particularly reliant on precise spatiotemporal control of RNA splicing for its development, plasticity, and homeostatic function [7]. Consequently, dysregulation of splicing mechanisms has been increasingly implicated in the pathogenesis of numerous neurodegenerative disorders. For instance, in Alzheimer's disease, aberrant splicing of the microtubule-associated protein tau contributes to the accumulation of pathogenic tau isoforms, while in Parkinson's disease, alternative splicing patterns of the α-synuclein are linked to disease risk and progression [8]. Notably, preclinical models have begun to illuminate a potential role for splicing dysregulation in PND, with observations of altered splicing activity in the hippocampus of aged mice following anesthesia and surgery correlating with subsequent cognitive deficits [9]. This emerging evidence positions RNA splicing as a compelling, though underexplored, molecular pathway in the pathogenesis of PND.

**Fig. 1:**
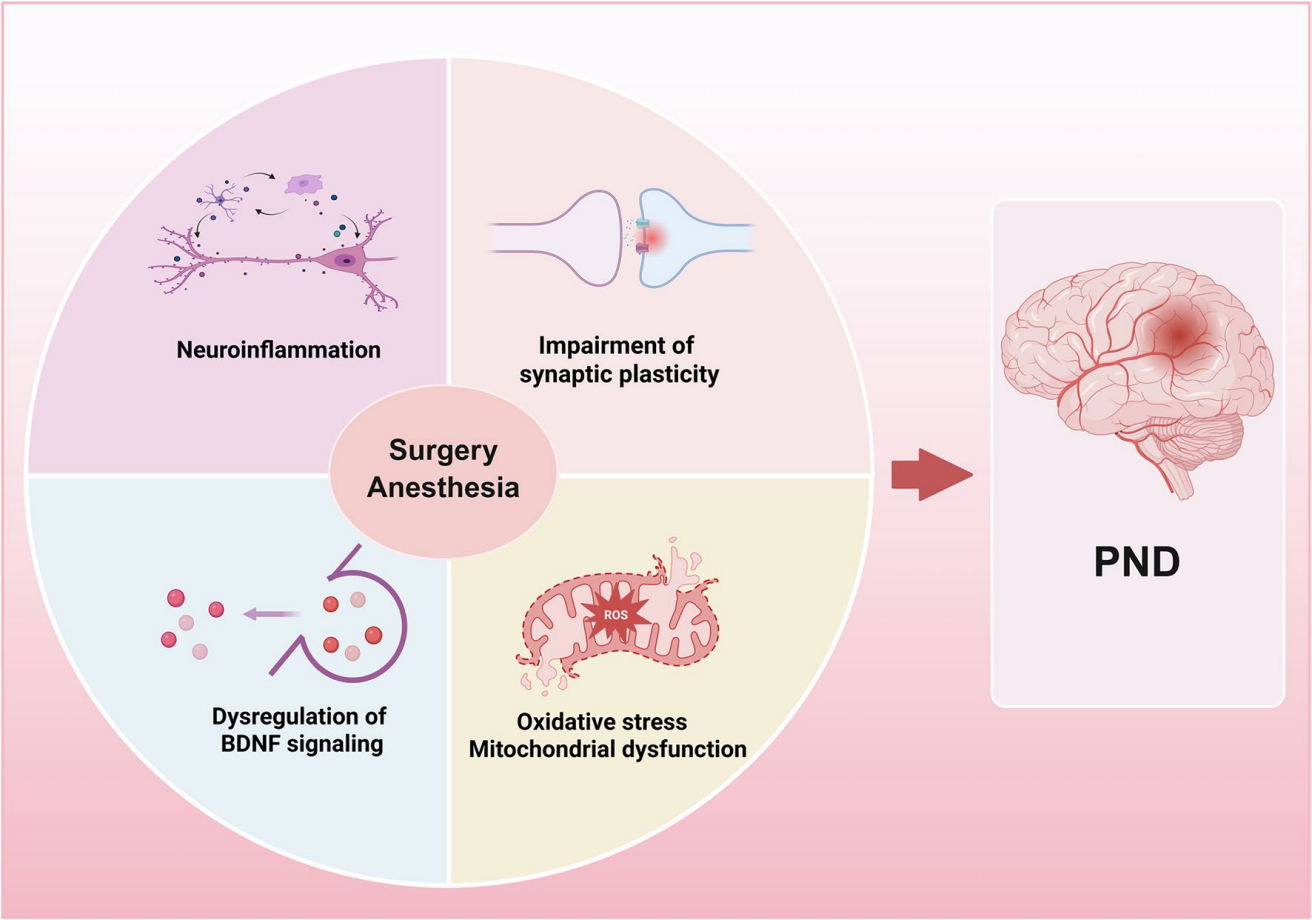
Mechanism of PND induced by surgery and anesthesia. Neuroinflammation, impairment of synaptic plasticity, dysregulation of BDNF signaling, oxidative stress, and mitochondrial dysfunction have been identified as closely related to the occurrence of PND. Abbreviations: PND, perioperative neurocognitive disorders; BDNF, brain-derived neurotrophic factor.

The established pathogenic mechanisms of PND are diverse and interrelated, prominently featuring neuroinflammation [10], impairment of synaptic plasticity [11], dysregulation of brain-derived neurotrophic factor (BDNF) signaling [12], oxidative stress [13], and mitochondrial dysfunction [14] (Fig. 1). This review article seeks to synthesize existing knowledge and explore the novel hypothesis that dysregulation of RNA splicing is an important regulatory factor that intersects with these canonical pathways. We will provide a comprehensive analysis of recent advances linking splicing fidelity to PND-related processes, critically evaluate the current limitations in the field, and discuss promising therapeutic strategies targeting splicing mechanisms. By elucidating the role of RNA splicing in PND pathogenesis, this review aims to identify key research gaps and outline future directions that could enhance our understanding and inform the development of targeted interventions for this debilitating condition.

## Dysregulation of RNA Splicing in Neuroinflammation

Neuroinflammation is a central pathological mechanism in the development of PND, contributing directly to neuronal damage and functional impairment through the synergistic actions of inflammatory cells and signaling molecules [15]. Surgical trauma, anesthetic agents, and postoperative stress can precipitate a cascade of events, including excessive cytokine release, glial cell activation, and blood-brain barrier disruption. These events facilitate the infiltration of peripheral immune cells into the central nervous system (CNS), amplifying the neuroinflammatory response [16]. The ensuing inflammation leads to the activation of microglia [17] and astrocytes [18] *via* multiple signaling pathways. Once activated, these glial cells release pro-inflammatory cytokines that promote cellular apoptosis and necroptosis, increase the accumulation of pathogenic proteins, and disrupt synaptic transmission, ultimately leading to neuronal injury [15]. Injured neurons can, in turn, further activate glial cells, establishing a self-perpetuating cycle that exacerbates PND progression [19]. Within this inflammatory milieu, RNA splicing emerges as a critical regulatory node. A growing body of evidence indicates that the dysregulation of splicing events can generate abnormal protein isoforms that fundamentally alter inflammatory processes within the CNS, highlighting a critical interaction between RNA splicing and neuroinflammation [20].

### Splicing Regulation of Inflammatory Mediators in PND

Dysregulated RNA splicing can profoundly affect neuroinflammation by altering the isoform expression of key inflammatory genes. For instance, the purinergic receptor P2X7, an adenosine triphosphate (ATP)-gated ion channel, regulates neuroinflammation by modulating the production of interleukin 1β (IL-1β) and IL-18, as well as by activating the NOD-like receptor protein 3 (NLRP3) [21, 22]. Alternative splicing of the P2X7 gene produces full-length (P2X7A) and truncated (P2X7B) variants, and a shift in this balance due to splicing dysregulation can impair pro-inflammatory signaling pathways by eliminating essential protein domains of P2X7 [23]. Similarly, deficiency in the kinase phosphatase and tensin homolog-induced kinase 1 (PINK1) can disrupt the expression of splicing factors like serine/arginine-rich splicing factor 10 (SRSF10), leading to an aberrant spliceosome that activates inflammatory pathways [24]. Furthermore, the long non-coding RNA (LncRNA) MALAT1 regulates splicing factors such as heterogeneous nuclear ribonucleoprotein F (hnRNP F), hnRNP H1, and SRSF1; its abnormal upregulation has been shown to alter the splicing of immune-related genes like interleukin 7 receptor (IL7R) and speckled protein 140 (SP140), thereby influencing the inflammatory response. This is supported by research in multiple sclerosis, where dysregulation of alternative splicing in genes such as IL7R has been linked to disease pathogenesis [25].

### Glial Splicing in Neuroimmune Activation

The activation and function of glial cells, which are fundamental to neuroinflammation, are also modulated by alternative splicing. The microglial receptor triggering receptor expressed on myeloid cells 2 (TREM2), which regulates inflammation, can be affected by genetic variants like the R47H mutation [26, 27]. This variant can induce aberrant splicing, leading to mRNA instability, reduced protein expression, and an increased risk of neurodegeneration. In astrocytes, inflammation can alter the splicing of the tyrosine-protein kinase Fyn, favoring the upregulation of the FynT isoform over FynB. The FynT variant has been specifically implicated in stimulating the synthesis of pro-inflammatory cytokines like IL-6 and IL-1β, thereby amplifying the neuroinflammatory cascade [28]. Additionally, this process can also be directly triggered by inflammatory cytokines such as tumor necrosis factor (TNF), which induces FynT expression *via* alternative splicing, leading to synaptic dysfunction and cognitive impairment [29].

### Inflammatory Feedback on Splicing Networks

Conversely, neuroinflammation itself is a potent regulator of RNA splicing patterns, creating a feedback loop that can either amplify or resolve the inflammatory response. A key study on a multiple sclerosis model demonstrated that inflammation in the prefrontal cortex alters the alternative splicing of neurexins 1-3 (NRXN1-3) at the alternative splice site 4 (AS4) site. This effect is mediated by the downregulation of the splicing factor Src-like molecule 2 (SLM2) in response to high IL-1β levels, leading to increased exon inclusion, changes in GABAergic circuitry, and cognitive dysfunction [30]. This illustrates how inflammatory signals can directly rewire the synaptic transcriptome through splicing regulation. Similarly, neuroinflammatory stimuli can modulate the expression of alternatively spliced isoforms of the chaperone protein resistance to inhibitors of cholinesterase-3 (RIC-3), which is critical for nicotinic acetylcholine receptor assembly. The shift between full-length and truncated RIC-3 isoforms can subsequently exert anti-inflammatory effects by modulating specific receptor subunits [31].

### Concluding Perspectives on RNA Splicing in Neuroinflammatory Pathways

**Fig. 2:**
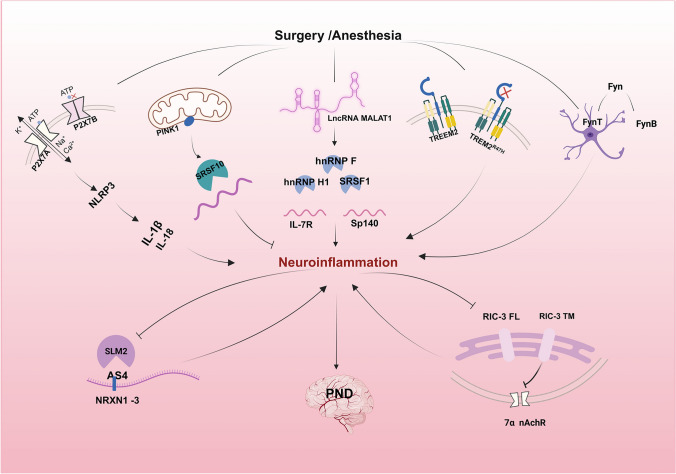
Dysregulation of RNA splicing in neuroinflammatory pathways contributes to PND. This schematic illustrates key molecular mechanisms through which aberrant RNA splicing modulates neuroinflammation. Specifically, alternative splicing of the P2X7 gene generates isoforms (e.g., P2X7A) that potentiate pro-inflammatory cytokine release. The kinase PINK1 regulates splicing factor SRSF10, whose dysregulation can promote inflammatory signaling. The LncRNA MALAT1 influences the activity of splicing regulators (e.g., hnRNP F, hnRNP H1, SRSF1), thereby affecting the splicing of immune-related genes such as IL7R and SP140, and exacerbating neuroinflammation. In microglia, the TREM2 R47H variant induces aberrant splicing, reducing TREM2 expression and increasing neuroinflammation risk. In astrocytes, inflammation-associated upregulation of the FynT splice variant enhances production of pro-inflammatory cytokines (e.g., IL-6, IL-1β). Conversely, neuroinflammatory signals can reciprocally alter splicing patterns: for example, neuroinflammation-induced downregulation of splicing factor SLM2 promotes inclusion of the AS4 exon in neurexin genes (NRXN1-3), contributing to cognitive decline. Additionally, inflammatory stimuli modulate splicing of the RIC-3 gene, reducing full-length isoform expression and attenuating anti-inflammatory signaling *via* nicotinic acetylcholine receptor α7. Abbreviations: PND, perioperative neurocognitive disorders; PINK1, PTEN-induced kinase 1; SRSF, serine/arginine-rich splicing factor; LncRNA, long non-coding RNA; hnRNP, heterogeneous nuclear ribonucleoprotein; IL, interleukin; SP140, speckled protein 140; TREM2, triggering receptor expressed on myeloid cells 2; SLM2, Src-like molecule 2; AS4, alternative splice site 4; NRXN1-3, neurexins 1-3; RIC-3, resistance to inhibitors of cholinesterase-3.

Overall, the significance of perioperative neuroinflammation in the onset and progression of PND is well-established, with central and peripheral inflammatory mechanisms implicated in the pathological process of PND (Table 1, Fig. 2). Emerging evidence indicates that RNA splicing serves as an important regulatory layer within this neuroinflammatory cascade. Dysregulation of splicing factors and specific splicing events can alter the expression profiles of key inflammation-related genes (e.g., those encoding cytokine receptors and immune regulators), thereby modulating microglial activation, cytokine production, and ultimately impacting neuronal homeostasis and cognitive function. Although preliminary findings imply a significant link between aberrant RNA splicing and PND pathogenesis, a more comprehensive understanding of the underlying molecular mechanisms—particularly regarding cell-type-specific splicing patterns and their functional consequences in the aged brain—is essential. Future investigations should aim to elucidate these pathways to assess their potential as novel diagnostic biomarkers or therapeutic targets for PND.

**Table 1:** Key molecules in RNA splicing-regulated neuroinflammation

Molecule	Type	Splicing mechanism	Neuroinflammatory function	References
P2X7	Ionotropic purinergic receptor	Alternative splicing (P2X7A/P2X7B)	P2X7A ↑→IL-1β, IL-18↑, NLRP3↑	[21, 22]
PINK1	Protein kinase	Regulates splicing factor (e.g., SRSF10)	Aberrant splice variants →inflammation	[24]
MALAT1	LncRNA	Regulates hnRNP F, hnRNP H1, SRSF1	MALAT1↑ →inflammation	[25]
TREM2	Immune regulatory receptor	R47H variant	R47H variant ↑ →Inflammation	[26, 27]
FynT	Non-receptor tyrosine kinase	Alternative splicing (FynB/FynT)	FynT ↑→IL-6/IL-1β↑ →impairs cognition	[28, 29]
NRXN1-3	Presynaptic adhesion molecule	Neuroinflammation → enhanced exon inclusion	Remodels GABAergic circuits → impairs cognition	[30]
RIC-3	Transmembrane protein	Neuroinflammation →Full-length RIC-3↓	Full-length RIC-3↓ → nAChR-α7↓ →anti-inflammation↓	[31]

IL, interleukin; NLRP3, NOD-like receptor protein 3; PINK1, PTEN-induced kinase 1; SRSF, serine/arginine-rich splicing factor; LncRNA, long non-coding RNA; hnRNP, heterogeneous nuclear ribonucleoprotein; TREM2, triggering receptor expressed on myeloid cells 2; NRXN1-3, neurexins 1-3; RIC-3, resistance to inhibitors of cholinesterase-3; nAChR, neuronal nicotinic acetylcholine receptor.

## Dysregulation of RNA Splicing in Impairment of Synaptic Plasticity

Synaptic plasticity, the ability of synapses to strengthen or weaken over time in response to activity, is a fundamental property of the nervous system essential for learning, memory, and adaptive behavior [32]. Long-term potentiation (LTP) and long-term depression (LTD) represent the principal cellular models of this plasticity, underlying cognitive processes such as memory formation and information filtering [33]. Impairments in synaptic plasticity are widely recognized as a core pathological mechanism in PND [34]. As a pivotal post-transcriptional mechanism, RNA splicing profoundly influences synaptic plasticity by generating diverse protein isoforms with distinct functions from a single gene [7]. This section examines how dysregulated RNA splicing potentially contributes to PND by disrupting the expression and function of key synaptic proteins.

### Regulation of Synaptic Protein Diversity by Alternative Splicing

Alternative splicing significantly expands the proteomic complexity critical for synaptic function. Presynaptic adhesion molecules such as NRXNs exemplify this regulation. The alternative splicing of NRXN3 at the SS4 site can modulate postsynaptic α-amino-3-hydroxy-5-methyl-4-isoxazolepropionic acid (AMPA) receptor trafficking, thereby influencing long-term plasticity [35]. Conversely, neuronal activity can drive splicing changes at the Nrxn1 exon 22 site, promoting NRXN1 SS4 inclusion and enhancing memory retention, illustrating an activity-dependent feedback loop [36]. Similarly, postsynaptic density protein 95 (PSD-95), a key scaffolding protein at excitatory synapses, is regulated by neuron-specific splicing. The inclusion of exon 18 in neurons ensures the production of functional PSD-95, whereas its exclusion in non-neuronal cells leads to nonsense-mediated decay, thereby restricting PSD-95 expression to neurons and supporting synaptogenesis [37]. Voltage-gated ion channels are also subject to precise splicing control. For instance, alternative splicing of the Cav2.1 gene generates two major P/Q-type voltage-gated calcium channel (VGCC) isoforms, Cav2.1[EFa] and Cav2.1[EFb], which exert opposing effects on synaptic efficacy. An upregulation of Cav2.1[EFa] in hippocampal neurons contributes to presynaptic homeostasis and facilitates synaptic plasticity [38].

### Splicing-Dependent Modulation of Neurotransmitter Receptors

RNA splicing critically shapes the functional properties of neurotransmitter receptors, thereby fine-tuning synaptic transmission. The N-methyl-D-aspartate (NMDA) receptor, crucial for synaptic plasticity, is a key target. The alternative splicing of exon 5 in the GluN1 subunit modulates receptor function; the absence of this exon has been shown to significantly enhance LTP in hippocampal synapses [39]. Beyond glutamate receptors, the integrity of the splicing machinery itself is vital. Dysfunction of the U1 small nuclear ribonucleoprotein (U1 snRNP), a core spliceosome component, can lead to widespread splicing errors. In murine primary neurons, U1 snRNP inhibition results in the downregulation of GABAergic synaptic components, linking splicing dysregulation directly to impaired inhibitory transmission and cognitive deficits [40].

### Activity-Dependent Feedback on RNA Splicing

The relationship between splicing and synaptic function is bidirectional, as neuronal activity can directly modulate splicing decisions. For example, neuronal excitation regulates the microexon splicing of eukaryotic translation initiation factor 4G (EIF4G), altering its interaction with the translational repressor fragile X mental retardation protein (FMRP) and splicing factors like RNA binding fox-1 (RBFOX) [41]. This mechanism influences the localized synthesis of synaptic proteins, thereby coupling synaptic activity to proteomic remodeling necessary for plasticity. Conversely, under conditions of chronic neuronal inactivity, calcium signaling pathways promote the nucleocytoplasmic shift of the RNA-binding protein neuro-oncological ventral antigen 2 (Nova2). This relocalization attenuates Nova2-dependent promotion of the large-conductance Ca^2+^-activated potassium channel (BK channel) E29 exon splicing, leading to prolonged action potentials—a homeostatic adaptation that maintains neuronal excitability [42].

### Conclusion and Therapeutic Implications on RNA Splicing in Impairment of Synaptic Plasticity

**Fig. 3:**
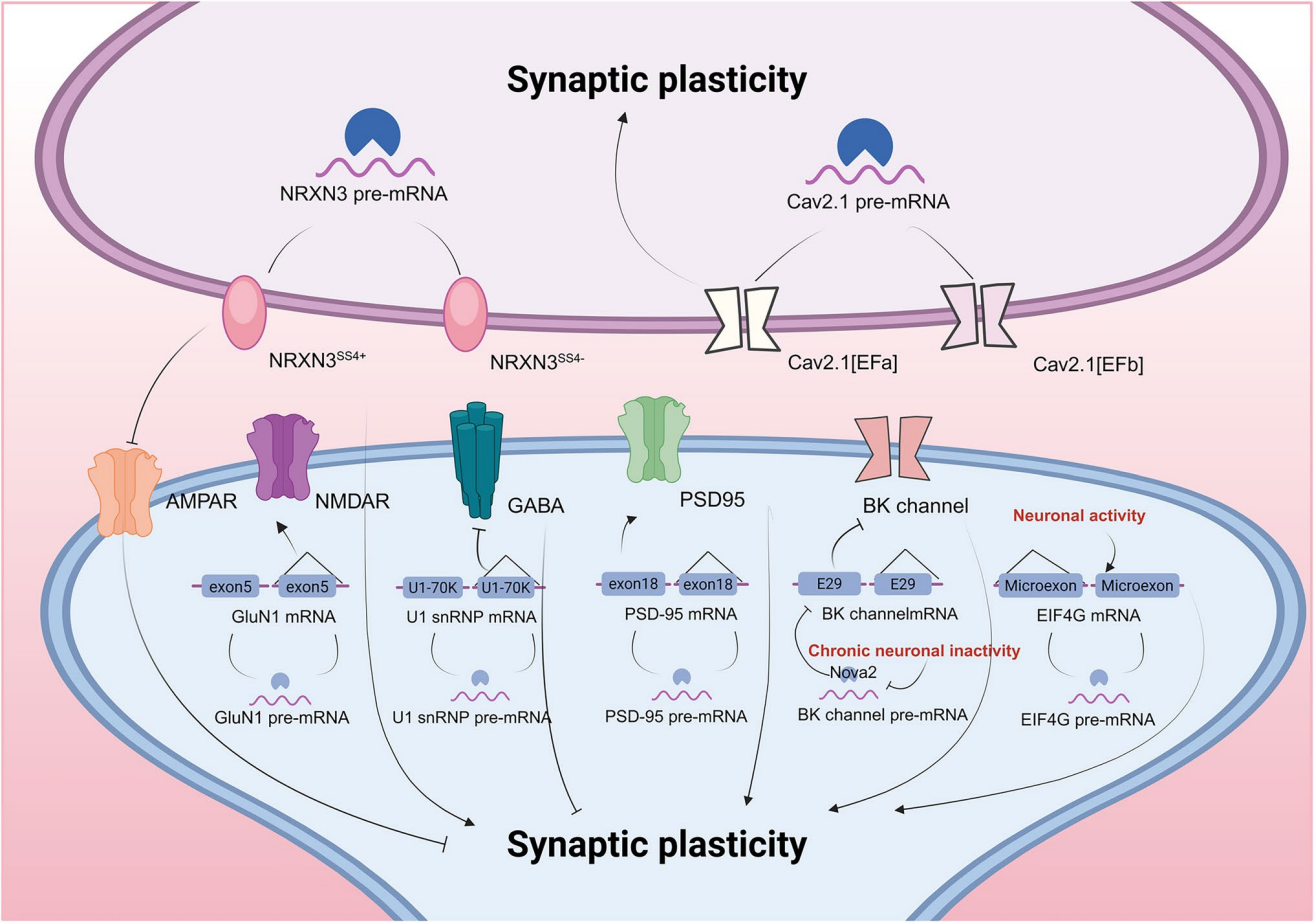
Dysregulation of RNA splicing contributes to synaptic dysfunction in PND. This schematic summarizes key molecular pathways through which aberrant RNA splicing impairs synaptic plasticity. Alternative splicing of presynaptic adhesion molecules such as NRXNs critically regulates synapse assembly and function. Inclusion of the alternative SS4 in NRXN3 disrupts the trafficking of postsynaptic AMPA receptors and suppresses LTP. VGCC Cav2.1 isoforms, generated through mutually exclusive splicing, exert opposing effects on synaptic efficacy: Cav2.1[EFa] enhances synaptic transmission and plasticity, whereas Cav2.1[EFb] attenuates it. Selective upregulation of Cav2.1[EFa] in hippocampal neurons contributes to presynaptic homeostasis. Splicing also governs the functional properties of neurotransmitter receptors. Exclusion of exon 5 in the GluN1 subunit of the NMDA receptor enhances LTP in hippocampal synapses. Conversely, dysfunction of the U1 snRNP complex, exemplified by reduced levels of its U1-70K core subunit, leads to widespread splicing errors and downregulation of GABAergic synaptic components, contributing to cognitive impairment. Neuron-specific inclusion of exon 18 in the PSD-95 transcript ensures proper protein synthesis and is essential for synapse development. Furthermore, splicing is dynamically modulated by neuronal activity. Electrical activity regulates the microexon splicing of EIF4G, influencing the synthesis of synaptic proteins and thereby affecting synaptic transmission and activity-dependent plasticity. Under conditions of chronic neuronal inactivation, the splicing factor Nova2 translocates from the nucleus to the cytoplasm, which inhibits the inclusion of the E29 exon in the large-conductance BK channel, leading to prolonged action potential duration as a homeostatic response. These mechanisms collectively highlight how splicing dysregulation disrupts synaptic proteostasis and neural circuit function, thereby promoting cognitive deficits in PND. Abbreviations: PND, perioperative neurocognitive disorders; NRXN, neurexin; SS4, alternative splicing site 4; AMPA receptor, α-amino-3-hydroxy-5-methyl-4-isoxazolepropionic acid receptor; LTP, long-term potentiation; VGCC, voltage-gated calcium channel; NMDA receptor, N-methyl-D-aspartate receptor; snRNP, small nuclear ribonucleoprotein; PSD-95, postsynaptic density protein 95; EIF4G, eukaryotic translation initiation factor 4G; Nova2, neuro-oncological ventral antigen 2; BK channel, Ca^2+^-activated potassium channel.

In summary, RNA splicing regulates synaptic plasticity through multiple convergent pathways: by generating diversity in synaptic adhesion molecules and ion channels, by modulating the subunit composition and function of neurotransmitter receptors, and by serving as a target for activity-dependent feedback regulation (Table 2, Fig. 3). Dysregulation within this intricate network may lead to the synaptic dysfunction observed in PND. Targeting these specific splicing anomalies presents a promising strategic avenue for developing interventions aimed at restoring synaptic homeostasis and improving cognitive outcomes following surgery.

**Table 2:** Key molecules in RNA splicing-regulated synaptic plasticity

Molecule	Type	Splicing mechanism	Synaptic plasticity	References
NRXN1	Presynaptic cell-adhesion molecule	NRXN1 SS4 inclusion	Memory retention ↑	[36]
NRXN3	Presynaptic cell-adhesion molecule	NRXN3 SS4 inclusion	Long-term plasticity ↓	[35]
PSD-95	Synaptic scaffold protein	Neuron-specific exon 18 inclusion	Synapse development ↑	[37]
Cav2.1 (VGCC)	Ca^2+^ channel α1 subunit	Mutually exclusive splicing (Efa/EFb isoforms)	Cav2.1[EFa] ↑ → synaptic efficacy ↑;	[38]
GluN1 (NMDAR)	NMDAR subunit	Alternative splicing of exon 5	Exon 5 exclusion ↑ →hippocampal LTP ↑	[39]
U1 snRNP	Splicing complex component	N40K overexpression displaces U1-70K → splicing dysfunction	GABAergic synaptic components ↓	[40]
EIF4G	Translation initiation factor	Neuronal activity regulates microexon splicing	Influences synaptic protein synthesis and plasticity	[41]
BK channel	Ca^2+^-activated K⁺ channel	Nova2-mediated E29 exon splicing	E29 splicing inhibition →action potential ↑	[42]

NRXN, neurexin; SS4, alternative splicing site 4; PSD-95, postsynaptic density protein 95; VGCC, voltage-gated calcium channel; NMDAR, N-methyl-D-aspartate receptor; LTP, long-term potentiation; snRNP, small nuclear ribonucleoprotein; N40K, N-terminal 40-kDa fragment; EIF4G, eukaryotic translation initiation factor 4G; BK channel, Ca^2+^-activated potassium channel; Nova2, neuro-oncological ventral antigen 2.

## Dysregulation of RNA Splicing in BDNF Signaling and Its Implications for PND

BDNF is a critical neurotrophin that supports neuronal survival, differentiation, and synaptic plasticity through its primary receptor, tropomyosin receptor kinase B (TrkB)[43]. It mediates both functional and structural plasticity within the central nervous system, influencing processes such as hippocampal neurogenesis and dendritic spine remodeling[44]. Clinical and preclinical studies indicate that reduced BDNF expression in the hippocampus and cortex correlates with cognitive impairment, highlighting its role in the pathophysiology of PND[11, 45]. Anesthesia and surgical stress can disrupt BDNF signaling, further supporting its involvement in PND[46]. The human BDNF gene exhibits considerable structural complexity, with multiple promoters and alternative splicing mechanisms generating numerous transcript isoforms. This intricate regulation allows for precise spatiotemporal control of BDNF expression in response to diverse physiological and pathological stimuli.

### Structural Complexity and Functional Diversity of BDNF Splice Variants

The BDNF gene comprises multiple 5' untranslated exons, each under the control of a distinct promoter, which are spliced to a common 3' coding exon (exon IX)[47]. This multi-promoter architecture enables nuanced regulation, as different promoters are activated by specific transcriptional factors and intracellular signaling cascades, such as those involving calcium influx [44]. Furthermore, the BDNF locus transcribes natural antisense RNAs (antiBDNF), which can form double-stranded structures with sense transcripts and influence BDNF pre-mRNA splicing and stability, adding another layer of post-transcriptional control [47].

Alternative splicing generates BDNF mRNA isoforms that differ primarily in their 5' untranslated regions (5'UTRs). These variations significantly impact the subcellular localization and translational efficiency of the transcripts. Specific 5'UTRs, such as those containing exon II or VI sequences, can target BDNF mRNA to dendrites, facilitating local protein synthesis in response to synaptic activity, which is crucial for sustained synaptic plasticity [48]. Additionally, certain 5' leader sequences can modulate BDNF protein synthesis by influencing translation initiation efficiency. Notably, utilization of the exon I-specific AUG yields higher BDNF protein levels than the conventional translation start site, attributable to enhanced overall translation efficiency [49].

### RNA-Binding Protein-Mediated Regulation of BDNF Splicing and Signaling

RNA-binding proteins (RBPs) serve as critical regulators of BDNF expression and function. The RBP RBFOX1 influences BDNF signaling by stabilizing the mRNA encoding a truncated TrkB isoform, TrkB.T1. This isoform acts in a dominant-negative manner, competing with the full-length TrkB receptor (TrkB.FL) for BDNF binding and impairing BDNF-dependent signaling cascades vital for synaptic plasticity [50]. The transcriptional regulator Methyl-CpG-binding protein 2 (MeCP2), mutations in which cause Rett syndrome, also plays a role in BDNF alternative splicing. MeCP2 functions in a complex with TET methylcytosine dioxygenase 1 (TET1) and the splicing factor Y-box binding protein 1 (YB-1) to modulate BDNF splicing. This complex orchestrates changes in DNA methylation and chromatin conformation at the BDNF locus, affecting the recruitment of splicing factors and fine-tuning BDNF isoform expression in a learning-dependent manner [51]. Furthermore, RBPs linked to neurodegeneration, such as FUS RNA-binding protein (FUS) and transactive response DNA-binding protein 43 (TDP-43), impact BDNF homeostasis. Mutations in FUS can lead to DNA damage accumulation and splicing defects that disrupt normal BDNF mRNA processing [52]. Similarly, TDP-43 dysfunction alters the splicing of Sortilin. As Sortilin is a key transport receptor for BDNF, aberrant splicing reduces BDNF secretion, thereby compromising synaptic plasticity and cognitive function [53].

### Activity-Dependent Splicing and Local Regulation of BDNF

BDNF pre-mRNA splicing is dynamically regulated by neuronal activity. Neuronal excitation can regulate the splicing of BDNF transcripts, such as those involving exon I, through calcium signals induced by L-type VGCCs [54]. This neuron-selective and activity-dependent splicing ensures efficient BDNF induction in response to synaptic demand. The regulation of BDNF localization and release is further fine-tuned by splicing events in genes encoding trafficking proteins. For instance, splice variants of the calcium-dependent activator protein for secretion 2 (Caps2) that lack exon 3 result in decreased BDNF release at axon terminals, highlighting how splicing directly influences the spatial control of BDNF signaling [55].

### Conclusion and Perspectives on RNA Splicing in BDNF Signaling

**Fig. 4:**
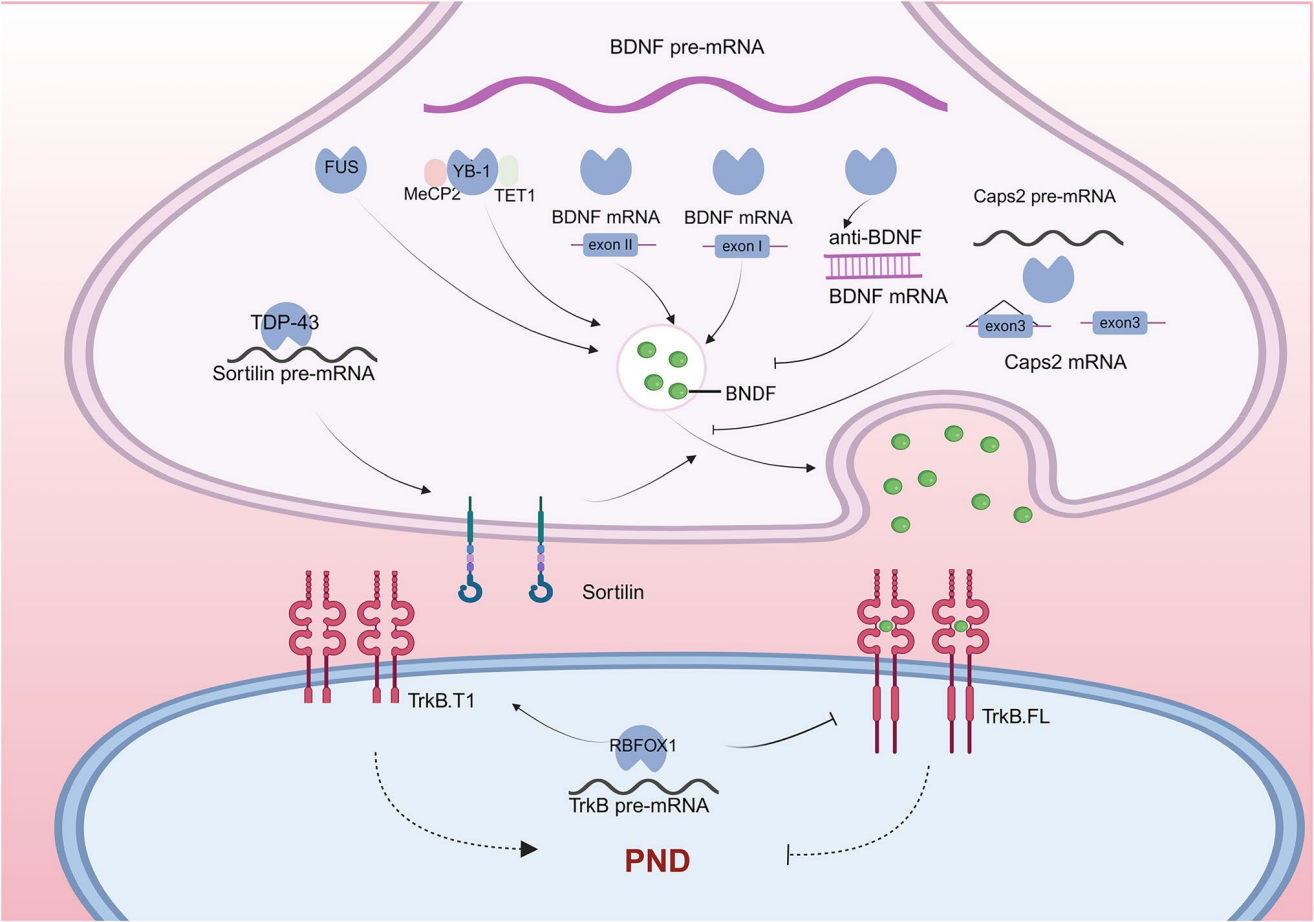
Mechanisms of RNA splicing-dependent regulation of BDNF in PND. The BDNF gene undergoes complex alternative splicing, generating multiple transcript isoforms with distinct 5′ untranslated regions that differentially influence mRNA localization, stability, and translation. Specific splice variants, such as those containing exon II, possess cis-regulatory elements that target BDNF mRNA to dendrites, facilitating local protein synthesis during synaptic activity. Variants containing exon I utilize an optimized translation initiation site, enhancing BDNF protein yield. Splicing factors, including FUS and TDP-43, regulate the processing of BDNF pre-mRNA, and their dysfunction can disrupt BDNF expression and promote neurodegeneration. The transcriptional regulator MeCP2, in a complex with TET1 and YB-1, modulates BDNF splicing and chromatin conformation in an activity-dependent manner, influencing cognitive processes. Natural antisense transcripts (antiBDNF) form RNA duplexes with BDNF mRNA, adding a layer of post-transcriptional control. Alternative splicing of the Caps2 influences dense-core vesicle trafficking, with exon 3-skipping variants reducing BDNF release at synaptic terminals. Furthermore, the splicing factor RBFOX1 stabilizes mRNA encoding a truncated BDNF receptor, TrkB.T1, which acts as a dominant-negative inhibitor of full-length TrkB signaling, thereby impairing LTP. Collectively, these splicing-mediated mechanisms fine-tune BDNF signaling, and their dysregulation may contribute to synaptic dysfunction and cognitive decline in PND. Abbreviations: BDNF, brain-derived neurotrophic factor; PND, perioperative neurocognitive disorders; FUS, FUS RNA-binding protein; TDP-43, transactive response DNA-binding protein 43; MeCP2, methyl-CpG-binding protein 2; TET1, TET methylcytosine dioxygenase 1; YB-1, Y-box binding protein 1; Caps2, calcium-dependent activator protein for secretion 2; RBFOX1, RNA-binding Fox-1 homolog 1; TrkB, tropomyosin receptor kinase B; TrkB.T1, truncated form of the BDNF receptor; LTP, long-term potentiation.

In summary, RNA splicing is a fundamental mechanism governing the expression, localization, and functional efficacy of BDNF (Table 3, Fig. 4). The generation of distinct BDNF splice variants, regulated by specific RNA-binding proteins and neuronal activity, ensures that BDNF signaling is optimally tuned to support synaptic plasticity and cognitive health. Dysregulation of these splicing mechanisms, potentially triggered by perioperative stressors, may disrupt BDNF homeostasis and induce synaptic dysfunction that is implicated in the pathological process of PND. A deeper understanding of these specific splicing pathways offers promising avenues for developing targeted therapeutic strategies aimed at preserving cognitive function following surgery.

**Table 3:** Key molecules in RNA splicing-regulated BDNF function

Molecule	Type	Splicing Mechanism	BDNF-Related Function	References
BDNF	Neurotrophin	11 exons generate splice variants; anti-BDNF modulates splicing	Variants control BDNF translation efficiency	[47–49]
RBFOX1	RNA-binding protein	TrkB.T1 mRNA ↑ → BDNF-TrkB binding↓	BDNF-mediated LTP ↓	[50]
MeCP2	Transcriptional regulator	Interacts with TET1 and YB1	BDNF splicing ↑	[56]
FUS	RNA-binding protein	FUS gene (e.g., R521C) mutation	Contributes to BDNF splicing defects	[57]
TDP-43	RNA-binding protein	Dysfunction alters Sortilin gene splicing	BDNF secretion ↓	[16]
Caps2	Secretory regulatory protein	Exon 3 deletion in splice variants	BDNF release at axon terminals ↓	[58]

BDNF, brain-derived neurotrophic factor; RBFOX1, RNA-binding Fox-1 homolog 1; TrkB, tropomyosin receptor kinase B; TrkB.T1, truncated form of the BDNF receptor; LTP, long-term potentiation; MeCP2, methyl-CpG-binding protein 2; TET1, TET methylcytosine dioxygenase-1; YB-1, Y-box binding protein 1; FUS, FUS RNA-binding protein; TDP-43, transactive response DNA-binding protein 43; Caps2, calcium-dependent activator protein for secretion 2.

## Dysregulation of RNA Splicing in Oxidative Stress and Mitochondrial Dysfunction

Oxidative stress arises from an imbalance between the production of reactive oxygen species (ROS) and the cell’s capacity to detoxify them, leading to disruption of redox homeostasis and damage to cellular components[56, 57]. Mitochondria are central to this process, as oxidative phosphorylation—while essential for ATP generation—also constitutes a primary source of endogenous ROS. Under physiological conditions, antioxidant systems maintain ROS within non-damaging levels; however, mitochondrial dysfunction can provoke excessive ROS generation while simultaneously impairing cellular defense mechanisms, thereby accelerating oxidative injury [58]. A self-perpetuating cycle is established: mitochondrial damage elevates ROS production, which in turn exacerbates mitochondrial impairment. In the perioperative context, surgical trauma, anesthesia, and systemic inflammatory responses can induce significant oxidative stress and mitochondrial dysfunction, contributing to neuronal injury, synaptic deficits, and neuroinflammation—key pathological features of PND [59]. Emerging evidence indicates that RNA splicing, a critical layer of post-transcriptional regulation, is highly sensitive to the cellular redox state and serves as both a target and amplifier of oxidative and mitochondrial stress pathways [60, 61].

### Splicing Regulation of Antioxidant Defense Systems​

The cellular antioxidant response is critically dependent on precise gene expression regulation, including alternative splicing, which generates functionally distinct protein isoforms. Splicing defects can alter the expression, activity, or localization of key antioxidant enzymes, thereby modulating oxidative stress susceptibility. For example, the RNA kinase cleavage and polyadenylation factor I subunit (CLP1), involved in tRNA and mRNA maturation, is essential for proper splicing fidelity. CLP1 mutations lead to the accumulation of unspliced tRNA precursors and sensitize cells to oxidative damage, illustrating how splicing machinery integrity supports redox homeostasis [62]. Similarly, the RNA-binding protein splicing factor proline and glutamine-rich (SFPQ), which often dimerizes with non-POU domain-containing octamer-binding protein (NONO), regulates splicing of transcripts involved in oxidative stress responses. Aberrant SFPQ splicing can disrupt adenosine-to-inosine (A-to-I) RNA editing patterns and compromise the expression of antioxidant enzymes, thereby weakening cellular defenses [63]. Furthermore, studies in neuronal cells show that mitochondrial toxins such as paraquat induce widespread alterations in alternative splicing patterns for genes encoding antioxidant and survival proteins, an effect attributed primarily to ATP depletion rather than oxidative damage per se[64]. This suggests that energy deficit during mitochondrial dysfunction may directly impair spliceosome function, leading to maladaptive splicing responses that further compromise antioxidant capacity.

### Splicing in Mitochondrial Integrity and Bioenergetics​

Nuclear-encoded mitochondrial proteins are frequently subject to alternative splicing, which fine-tunes their expression and function. Disruption of this regulation can directly impair mitochondrial quality and energy production. For instance, aberrant splicing of NADH dehydrogenase ubiquinone iron-sulfur protein 6 (NDUFS6), a component of mitochondrial complex I (CI), due to mutations such as c.309+5G>A, generates defective isoforms that disrupt complex assembly, reduce electron transport efficiency, and promote ROS leakage [65]. Similarly, the mitochondrial fission factor dynamin-related protein 1 (Drp1) undergoes alternative splicing of exons A, B, C, and D, producing isoforms with distinct subcellular localization and function. Altered Drp1 isoform ratios perturb mitochondrial dynamics, impair respiratory function, and have been linked to neuronal vulnerability [14, 66]. Such splicing-related disturbances in mitochondrial proteome integrity may substantially contribute to the energy insufficiency and oxidative stress burden implicated in PND.

### Splicing-Feedback Loops Between Oxidative Stress and Mitochondrial Damage​

A vicious cycle can arise wherein mitochondrial dysfunction and oxidative stress mutually reinforce splicing defects, amplifying cellular injury. On one hand, ROS-induced oxidation of splicing factors—such as TDP-43 or FUS—can alter their RNA-binding affinity, subcellular localization, or protein-protein interactions, leading to erroneous splicing of transcripts encoding mitochondrial and antioxidant proteins. For example, TDP-43 depletion or mutation causes mis-splicing of mitofusin 2 (MFN2) (involved in mitochondrial fusion) and thioredoxin-interacting protein (TXNIP) (a negative regulator of antioxidant signaling), thereby disrupting mitochondrial dynamics and enhancing oxidative stress [67, 68]. On the other hand, oxidative modification of TDP-43 itself promotes its aggregation and functional impairment, further distorting the transcriptome [69]. This reciprocal disruption creates a pathogenic feedback loop that accelerates neuronal dysfunction. Similar dysregulation is observed in other neurodegenerative contexts; in amyotrophic lateral sclerosis (ALS) models, mutations in C9orf72 lead to toxic dipeptide repeats that sequester splicing factors, causing broad splicing abnormalities and mitochondrial defects [70]. These findings highlight that splicing dysregulation can act as both a cause and a consequence of oxidative and mitochondrial stress, forming a reinforcing loop that may promote PND progression.

### Conclusion and Therapeutic Outlook on RNA Splicing in Oxidative Stress and Mitochondrial Dysfunction

**Fig. 5:**
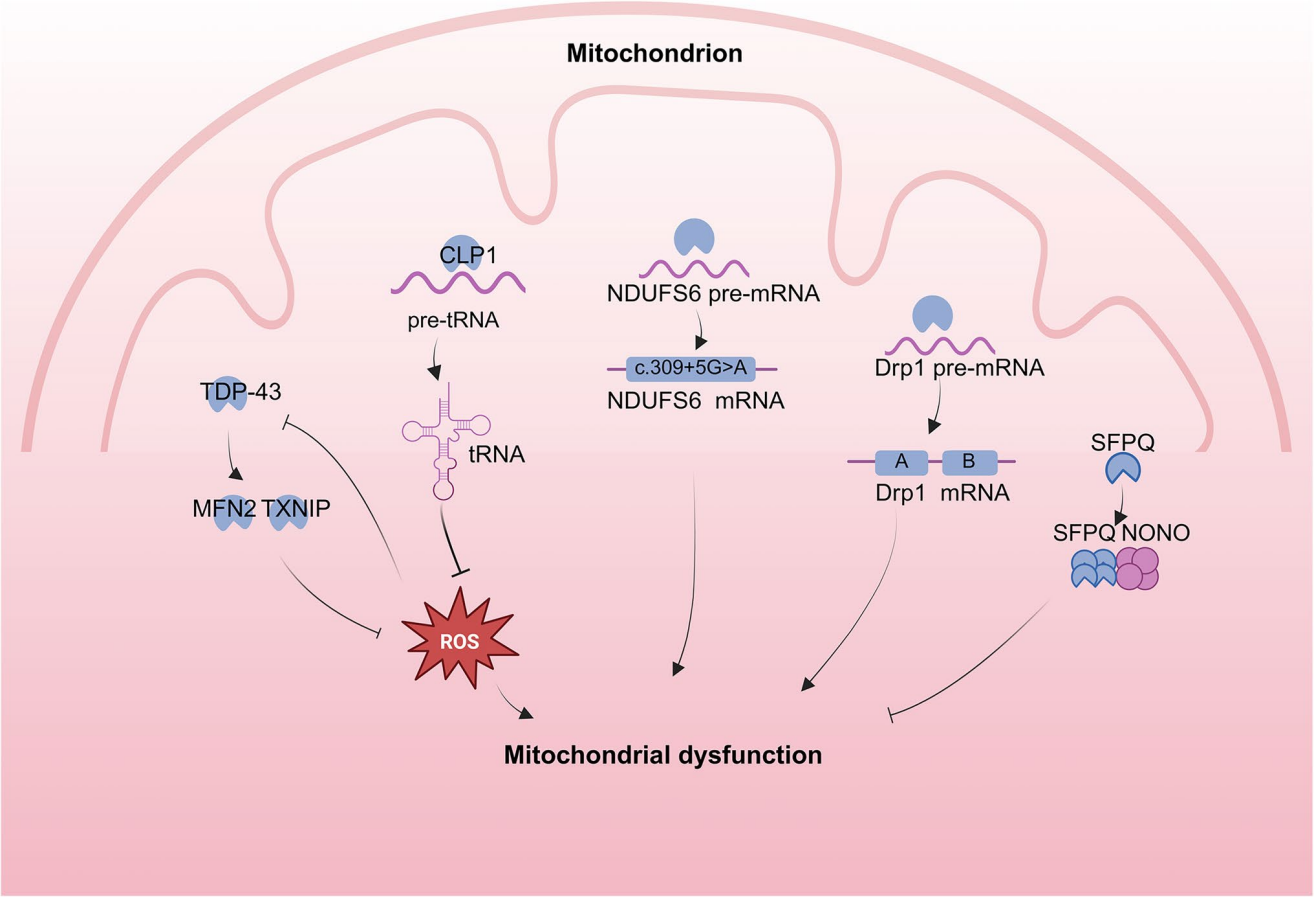
Mechanisms of RNA splicing dysregulation in oxidative stress and mitochondrial dysfunction contributing to PND. This schematic illustrates how aberrant RNA splicing impacts cellular redox homeostasis and mitochondrial integrity, key pathways implicated in PND pathogenesis. Mutations in the tRNA splicing kinase CLP1 disrupt tRNA maturation, leading to accumulation of unspliced tRNA precursors and heightened oxidative damage. Impaired splicing of the mitochondrial complex I subunit NDUFS6 generates defective isoforms that disrupt electron transport chain assembly, compromising ATP production and promoting mitochondrial dysfunction. Alternative splicing of Drp1 produces isoforms (e.g., those containing exons A/B) with distinct subcellular localizations and functions; aberrant Drp1 isoform ratios perturb mitochondrial fission, dynamics, and interactions with lysosomes, further impairing bioenergetics. The RNA-binding protein SFPQ, when properly spliced, promotes A-to-I RNA editing and supports the expression of antioxidant enzymes, enhancing cellular defense against oxidative stress. Conversely, loss of TDP-43 function or its oxidative modification causes mis-splicing of mitochondrial genes (e.g., MFN2 and TXNIP), resulting in excessive ROS production; this oxidative stress further impairs TDP-43 function, creating a self-amplifying cycle of splicing defects and mitochondrial damage. Collectively, these splicing anomalies disrupt the redox balance and mitochondrial proteome, driving neuronal vulnerability and cognitive decline in PND. Abbreviations: PND, perioperative neurocognitive disorders; CLP1, cleavage and polyadenylation factor I subunit; NDUFS6, NADH dehydrogenase ubiquinone iron-sulfur protein 6; ATP, adenosine triphosphate; Drp1, dynamin-related protein 1; SFPQ, splicing factor proline and glutamine rich; NONO, non-POU domain-containing octamer-binding protein; A-to-I, adenosine-to-inosine; TDP-43, transactive response DNA-binding protein 43; MFN2, mitofusin 2; TXNIP, thioredoxin-interacting protein; ROS, reactive oxygen species.

In summary, RNA splicing may represent an important mechanistic link connecting oxidative stress and mitochondrial dysfunction in PND pathogenesis (Table 4, Fig. 5). Splicing abnormalities disrupt the expression of antioxidant enzymes and mitochondrial proteins, while oxidative and energetic stress directly impair splicing fidelity. This reciprocal relationship fuels a self-amplifying cycle of cellular damage that is implicated in the pathological process of PND. Targeting these interactions—for instance, by correcting specific mis-splicing events, enhancing spliceosome function under stress, or mitigating oxidative RNA damage—offers promising avenues for therapeutic intervention. A deeper understanding of how splicing programs are rewired under perioperative stress will not only clarify PND mechanisms but also identify novel biomarkers and precision treatments aimed at preserving cognitive function after surgery.

**Table 4:** Key molecules in RNA splicing-regulated oxidative stress and mitochondrial function

Molecule	Type	Splicing mechanism	Functional consequence	References
CLP1	Multifunctional kinase	Mutations disrupt tRNA splicing	Oxidative stress ↑	[62]
SFPQ	RNA-binding factor	Aberrant splicing → abnormal A-to-I editing	Antioxidant capacity ↓	[63]
NDUFS6	Mitochondrial protein	c.309+5G>A variant	Impairs mitochondrial CI assembly	[65]
Drp1	Dynamin-related GTPase	Alternative splicing (exons A-D)	Aberrant isoforms ratio → mitochondrial fission	[14, 66]
TDP-43	RNA-binding splicing factor	TDP-43 deficiency →MFN2/TXNIP splicing defects	Disrupts mitochondrial function	[68–70]
C9orf72	DENN domain-containing protein	Mutations in C9orf72 →Sequestration of splicing factors	Mitochondrial defects	[71]

CLP1, cleavage and polyadenylation factor I subunit; SFPQ, splicing factor proline and glutamine-rich; NDUFS6, NADH dehydrogenase ubiquinone iron-sulfur protein 6; CI, complex I; Drp1, dynamin-related protein 1; TDP-43, transactive response DNA-binding protein 43; MFN2, mitofusin 2; TXNIP, thioredoxin-interacting protein

## Overcoming PND-Specific Translational Barriers

### Research Limitations

Despite the growing insights into the association between RNA splicing dysregulation and PND pathogenesis, the current body of research is subject to several notable limitations that need to be acknowledged and addressed in future investigations.

First, the existing evidence is predominantly indirect and correlative rather than causal. Most studies linking RNA splicing to PND pathophysiology rely on extrapolation from mechanistic findings in other neurodegenerative diseases, such as Alzheimer’s disease, Parkinson’s disease, and multiple sclerosis. Direct experimental validation of the causal role of aberrant RNA splicing in PND-specific models and clinical samples remains scarce, and the specificity of these splicing-related mechanisms to PND has not been fully elucidated.

Second, the preclinical research models employed to study RNA splicing in PND have inherent limitations. Most studies are based on young or aged murine models induced by a single anesthetic agent or surgical procedure, which fail to recapitulate the complex clinical context of PND in humans. The human perioperative setting involves a synergistic combination of risk factors, including advanced age, pre-existing comorbidities, surgical trauma, prolonged anesthesia exposure, and perioperative systemic inflammation, and current animal models lack the ability to simulate this multi-factor pathological microenvironment, leading to a gap between preclinical findings and clinical reality.

Third, there is a critical paucity of clinical evidence supporting the role of RNA splicing in PND. To date, no large-scale transcriptomic studies have been conducted on human PND patients to identify characteristic profiles of RNA splicing dysregulation in central nervous system tissues or peripheral biofluids. Additionally, there is a lack of clinical cohort studies validating the correlation between specific splicing factors, aberrant splice isoforms, and the occurrence, severity, and long-term prognosis of PND, which hinders the clinical translation of splicing-related research findings into diagnostic or prognostic biomarkers.

Fourth, the mechanistic understanding of RNA splicing in PND pathogenesis remains incomplete and superficial. Current research has only preliminarily clarified the unidirectional association between RNA splicing dysregulation and core pathological processes of PND, such as neuroinflammation, synaptic plasticity impairment, BDNF signaling dysfunction, oxidative stress, and mitochondrial dysfunction. However, the bidirectional feedback regulation between PND pathological microenvironment and RNA splicing machinery has not been fully explored, especially the cell-type-specific splicing patterns and functional consequences in neurons, microglia, and astrocytes under perioperative stress. Furthermore, the upstream signaling pathways that trigger splicing factor dysregulation and the downstream molecular cascades mediated by aberrant splice isoforms in PND are yet to be systematically elucidated.

### Translational Barriers

While the therapeutic potential of targeting RNA splicing in PND is compelling, successful clinical translation faces several unique, disorder-specific challenges that must be strategically addressed. Future research must develop solutions for these translational barriers.

First, the timing of therapeutic intervention​ is critical. The perioperative period represents a narrow, well-defined window for intervention. Prophylactic strategies aimed at preventing the initiation of splicing dysregulation may be more effective than treatments administered after cognitive decline is manifest. However, this requires predictive biomarkers to identify high-risk patients preoperatively. Research should focus on validating splicing-related biomarkers in easily accessible samples, such as blood or exosomes, that correlate with central nervous system changes and PND susceptibility. This would enable a precision medicine approach where interventions are tailored to at-risk individuals, maximizing efficacy and minimizing unnecessary exposure.

Second, the blood-brain barrier (BBB)​ presents a formidable obstacle for delivering splicing-modulating therapeutics, such as antisense oligonucleotides (ASOs) or small-molecule modulators. Systemic administration may require carriers that facilitate BBB penetration. Promising strategies include the development of novel delivery systems. For instance, leveraging receptor-mediated transcytosis by conjugating therapeutics to ligands for BBB-specific transporters (e.g., transferrin receptor) could enhance brain uptake. Alternatively, exosomes or lipid nanoparticles engineered to target brain endothelium offer a promising avenue for non-invasive delivery. The efficacy of intraventricular or intrathecal administration of ASOs, successful in other neurological disorders, should also be explored in PND models, though the requirement for specialized delivery is a significant clinical hurdle.

Third, the heterogeneity of PND pathogenesis​ implies that a single splicing target may not be effective for all patients. The molecular drivers of PND may vary based on patient age, genetic background, surgical type, and pre-existing neuroinflammation or neurodegenerative pathology. Therefore, a multi-target approach or the development of interventions that modulate key upstream regulators of splicing (e.g., core spliceosome components or major stress-sensing kinases) might yield broader efficacy. However, this must be balanced against the risk of off-target effects. Utilizing advanced delivery systems such as the pH-sensitive, tumor-targeting gene carrier FA-PEG-CCTS/PEI/NLS/pDNA (FPCPNDs)[71], which demonstrates efficient cellular uptake and endosomal escape, provides a conceptual framework for designing sophisticated, PND-specific delivery platforms that could be adapted for splice-correcting therapies.

Finally, the safety profile of any intervention must be exceptionally high for an elective surgical population. Strategies that temporarily modulate splicing, rather than cause permanent genomic changes, are preferable. The transient nature of the perioperative insult suggests that a short-term intervention could be sufficient to prevent the cascade leading to persistent cognitive deficits. Research should prioritize therapeutics with reversible mechanisms and short half-lives.

In conclusion, while the path is complex, overcoming these barriers—through the development of predictive biomarkers, advanced BBB-penetrating delivery systems, and transient, safe therapeutic modalities—is essential for harnessing the power of RNA splicing modulation to improve cognitive outcomes for the millions of patients undergoing surgery annually.

## Prospects and Future Research Directions

Despite considerable progress in defining the contribution of RNA splicing dysregulation to PND, significant knowledge gaps persist. A primary limitation is the heavy reliance on in vitro models and animal studies, which may not fully recapitulate the intricate pathophysiology of human PND, including the complex interplay of aging, comorbidities, and the systemic stress response to surgery and anesthesia. Consequently, the clinical translatability of many mechanistic findings remains uncertain. Furthermore, the specific trans-acting splicing factors and cis-regulatory elements that constitute the aberrant splicing networks in PND are inadequately characterized. The mechanistic links connecting splicing errors to downstream pathways—such as neuroinflammation, synaptic dysfunction, BDNF signaling deficits, oxidative stress, and mitochondrial dysfunction—also require deeper elucidation. Importantly, the interplay between splicing regulation and other molecular processes, including epigenetic modifications and post-translational protein regulation, is largely unexplored in the perioperative context. Finally, a critical paucity of longitudinal clinical studies has hindered the validation of splicing-related biomarkers or their correlation with long-term cognitive outcomes in surgical patients.

Translational and clinical applications of RNA splicing research in PND are of high clinical value and represent key exploration directions, in line with the core mechanistic and clinical needs of PND management. Splicing-based biomarkers (e.g., aberrant splice isoforms of P2X7, BDNF, and other PND-pathogenic genes) enable minimally invasive, objective early diagnosis and prognosis of PND *via* peripheral biofluid detection. Multiplexed biomarker panels can further enhance diagnostic accuracy by addressing the pathogenic heterogeneity of PND. Perioperative risk stratification for PND can be significantly optimized by integrating preoperative splicing profiles (baseline splicing signatures and anesthetic-specific splicing responses) with traditional clinical risk factors. This integrated hybrid model can identify high-risk patients and guide personalized perioperative interventions, such as tailored anesthetic selection and targeted prophylaxis. Therapeutic modulation of splicing mechanisms provides disease-modifying strategies for PND. These strategies include precision splice correction using BBB-penetrant ASOs that target core pathogenic splicing events, perioperative administration of orally bioavailable small-molecule splicing factor modulators with transient activity to avoid long-term off-target effects, and exosome-mediated delivery of functional splicing factors to restore CNS splicing homeostasis in patients with severe splicing dysregulation.

Future research should address the aforementioned knowledge gaps through targeted approaches. First, there is an urgent need for comprehensive molecular profiling of RNA splicing events in human PND. Leveraging advanced transcriptomic technologies, such as single-cell and long-read sequencing, on patient-derived biospecimens will enable the discovery of novel splicing variants and the precise mapping of splicing factor dysregulation associated with cognitive decline. Second, research must delve deeper into the mechanistic pathways, particularly elucidating how perioperative stressors (e.g., inflammatory cytokines, anesthetic agents) serve as upstream signals that disrupt the splicing machinery in vulnerable cell types (e.g., microglia, neurons). A promising approach involves using clustered regularly interspaced short palindromic repeats (CRISPR)-based screening or chemical biology tools to identify key splicing factors whose modulation can attenuate neuronal damage [72]. Third, the therapeutic potential of rectifying aberrant splicing merits rigorous investigation, particularly the development and evaluation of novel blood-brain barrier (BBB)-optimized antisense oligonucleotides (ASOs) and small-molecule splicing modulators, which possess the advantageous properties of oral bioavailability and broad tissue distribution. Finally, design multi-center longitudinal clinical trials to validate splicing-based biomarkers for PND risk stratification, and evaluate the safety and efficacy of splicing-targeted therapeutics in high-risk surgical populations with short-term (postoperative delirium) and long-term (persistent cognitive decline) clinical endpoints.

In summary, clarifies that RNA splicing is a key regulatory mechanism involved in PND pathogenesis, and its potential as an upstream pathogenic factor remains to be further confirmed by in vivo and clinical studies, with pleiotropic regulatory effects on the core pathological processes of PND. A sophisticated understanding of the splicing code that governs neuronal homeostasis in the perioperative period will provide fundamental insights into PND pathogenesis. By addressing the existing research gaps through integrated multi-omics, mechanistic studies, and therapeutic innovation, the field can pave the way for groundbreaking strategies to preserve cognitive function and improve the quality of life for the growing number of patients undergoing surgical procedures.
